# Potentially Hazardous Drug-Drug Interactions Associated With Oral Antineoplastic Agents Prescribed in Chinese Tertiary Care Teaching Hospital Settings: A Multicenter Cross-Sectional Study

**DOI:** 10.3389/fphar.2022.808848

**Published:** 2022-02-01

**Authors:** Haitao Wang, Haitao Shi, Yan Wang, Na Wang, Youjia Li, Qianting Yang, Ya Li, Chenwei Liu, Ying Zan, Siping Feng, Jiao Xie

**Affiliations:** ^1^ Department of Pharmacy, The Second Affiliated Hospital of Xi’an Jiaotong University, Xi’an, China; ^2^ Department of Gastroenterology, The Second Affiliated Hospital of Xi’an Jiaotong University, Xi’an, China; ^3^ Department of Medical Oncology, The Second Affiliated Hospital of Xi’an Jiaotong University, Xi’an, China; ^4^ Department of Pharmacy, Yan’an People’s Hospital, Yan’an, China

**Keywords:** oral antineoplastic agents, cancer, drug-drug interaction (DDI), prevalence, adverse drug events (ADE)

## Abstract

**Background:** Oral administration increases the risk of interactions, because most oral antineoplastic agents (OAAs) are taken on a daily basis. Interactions can increase exposure to antitumoral agents or cause treatment failure. Potential drug–drug interactions (DDIs) are commonly observed in patients with cancer, while the extent to which OAAs related hazardous DDIs remains unclear.

**Methods:** We studied the contraindication patterns between oral antineoplastic agents and other medications among cancer patients in two tertiary care teaching hospitals in China. A total of 20 clinically significant hazardous DDI pairs that involved 30 OAAs were identified based on the predetermined criteria. Patient medications were checked for DDIs by using the US Food and Drug Administration approved labeling. Descriptive statistics and uni- and multivariate logistic regression analyses were carried out.

**Results:** In this study, 13,917 patients were included and a total of 297 DDIs were identified. The results revealed that proton pump inhibitors (PPIs), dexamethasone and fluoroquinolones were the most often involved hazardous DDIs with OAAs. The most prevalent contraindication is the simultaneous use of certain molecular targeted agents and PPIs. In the result of the multivariate analysis, younger age (0–20 group), increasing number of drugs and patient treated with targeted therapy had a higher risk for DDIs.

**Conclusion:** The prevalence of OAAs related hazardous DDIs appears to be low in the cancer patients. However, physicians and clinical pharmacologists should be aware of the potential hazardous DDIs when prescribing OAAs, especially certain pH-dependent molecular targeted agents and potential QTc prolonging drugs.

## Introduction

Cancer patients are at a higher risk of drug-drug interactions (DDIs), because they usually take multiple medications. Moreover, the majority of cancer patients are elderly individuals who require additional medications due to comorbidities (Riechelmann et al., 2007; Targownik et al., 2007; Shinohara et al., 2018; Herrmann, 2020). Studies have shown that 0.6–5% of hospitalizations related to adverse drug events (ADEs) are because of drug interactions, which is generally believed to account for a significant proportion of ADEs (Becker et al., 2007; Roughead et al., 2010). Additionally, approximately 4% of cancer-related deaths were assessed to be brought about by DDIs (Buajordet et al., 2001). DDIs involving oral anticancer therapies may reduce the effectiveness or increase the risk of toxicities resulting in unexpected treatment outcomes (Riechelmann et al., 2007).

A risky DDIs would have a negative impact on most patients taking the combination, or the risks far outweigh the potential benefits. Cancer patients in hospital settings are usually treated with multidrug regimens that increase the likelihood of dangerous interactions when anticancer drugs are needed. A review showed that 34% of cancer patients treated with chemotherapy have experienced at least one severe DDIs (defined as life-threatening or irreversible damage) that can have serious clinical consequences (van Leeuwen et al., 2011). The increased burden of treatment for cancer patients increases the risk of such prescriptions. Therefore, prevention of adverse interactions is one of the most important factors to consider in terms of efficacy and safety when deciding on drug prescription (Chen et al., 2005). Although several studies have evaluated the prevalence of potential drug interactions in cancer patients (Riechelmann et al., 2007; van Leeuwen et al., 2011), the prevalence of potential DDIs associated with oral antineoplastic agents (OAAs) is unknown. We systematically analyzed the risk patterns between OAAs and other medications that occurred over a three-year period (2018–2020) to determine whether these warnings were contraindicated according to drug labeling in the Chinese hospitals.

## Materials and Methods

A two-center cross-sectional study of contraindications patterns between OAAs and other patient medications was conducted in 2018–2020 for all cancer patients in the two Chinese tertiary care hospitals (The Second Affiliated Hospital of Xi’an Jiaotong University and Yan’an People’s Hospital). Computerized drug interaction screening programs do not exist in either hospital. The study protocol was approved by the ethics committees of both hospitals. All medical data were collected from the electronic hospital data management system of each selected hospital from January 2018 to December 2020.

All patients were treated with OAAs in the hospital settings. Analysis was limited only to patients who received two or more than two drugs where at least one of them was oral antineoplastic agents (Supplementary Table S1). Potentially hazardous drug interactions that did not include oral chemotherapy were excluded from the study. We also removed interactions with products that were not identifiable from the hospital’s electronic data, such as herbal medicines, over-the-counter drugs, alcohol, orange and grapefruit juices, and the topical medications for skin conditions. The hazardous drug combinations were tested when the exposure period for two medicines was matched. We defined co-prescribing as one or more overlapping days of supply between the OAAs and DDI drugs. If a drug contained more than one pharmacologically active ingredient, each drug was counted separately in the analysis. If a patient took the same drug in two or more formulation (e.g., i.v. or oral moxifloxacin for infections control), the drug was counted only once.

An initial list of hazardous combinations of any oral antineoplastic agent with any medication was checked for DDIs using the Micromedex drug–drug interaction program (Buajordet et al., 2001; Micromedex Solutions). DDI pairs were considered clinically important in the study if they were listed on Micromedex with a justification assessment that was established or may be contraindicated, major, or moderate. After extensive screening of drug information software, the Food and Drug Administration (FDA)-approved product labeling was used to determine the final list of hazardous combinations as a second screening. The terms “contraindicated”, “avoid” or “do not be co-administered” were used to identify the presence of hazardous drug-drug interactions within the FDA-approved label. Only the specific drugs listed on the FDA-approved label were used to identify the DDI pair in the analysis. OAAs have been defined as all cytostatic, antihormonal and molecular targeted drugs to treat malignancies. The hazardous combinations were counted only once when patients were given the same type of drug in succession, since the interaction mechanism of these drugs on oral antineoplastic agents is same, such as lansoprazole and omeprazole. DDIs can be categorized by mechanism in two major groups: pharmacokinetic DDIs and pharmacodynamic DDIs.

Descriptive statistics were applied to characterize the entire study sample in terms of demographics, cancer type, length of hospital stay, type of anticancer agents, comorbidities, number of drugs per patient, and interaction characteristics. Patients were divided into five age groups: pediatric (0–20 years old), 21–40 years old, 41–60 years old, 61–80 years old, and over 81 years old. Univariate and multivariate logistic regression analyzes were performed to identify the potential risk factors for the occurrence of contraindicated interactions. The occurrence of at least one potentially adverse interaction per patient was the dependent variable. And the explanatory variables were age, number of drugs, number of comorbidities, type of tumor (haemato-oncology/oncology), length of hospitalization and type of treatment. Gender was not included as a covariate variable due to the fact that certain types of cancer are only found in males or females. For binary or nominal variables, the lower risk group was chosen as the referent. Variables with univariate *p* values < 0.05 were included in the multivariate analysis. In the multivariate analysis, a *p* value of <0.05 was considered statistically significant.

## Results

Overall, a total of 13,917 patients were investigated during the 3-years study period, with a mean age of 57 years (range 4–95 years) and 9,301 patients (66.8%) were female. The median number of drugs used per person was 7 (range 1–63 drugs) and the median hospital stay was 5 days (range 1–90 days). The median number of comorbidities per patient was 0.5 (range 0–8) and 30.1% of all patients had at least one comorbidity. Demographic characteristics are listed in Supplementary Table S2. To evaluate drug interactions, the drugs as a victim drug and perpetrator drug were included both in the analysis, but no contraindicated DDIs for the perpetrators were identified. The antineoplastic agents used as the victim drugs were not strong CYP3A4 inducers or inhibitors and therefore had no significant effect on other drugs.

### Drug–Drug Interactions

**FIGURE 1:**
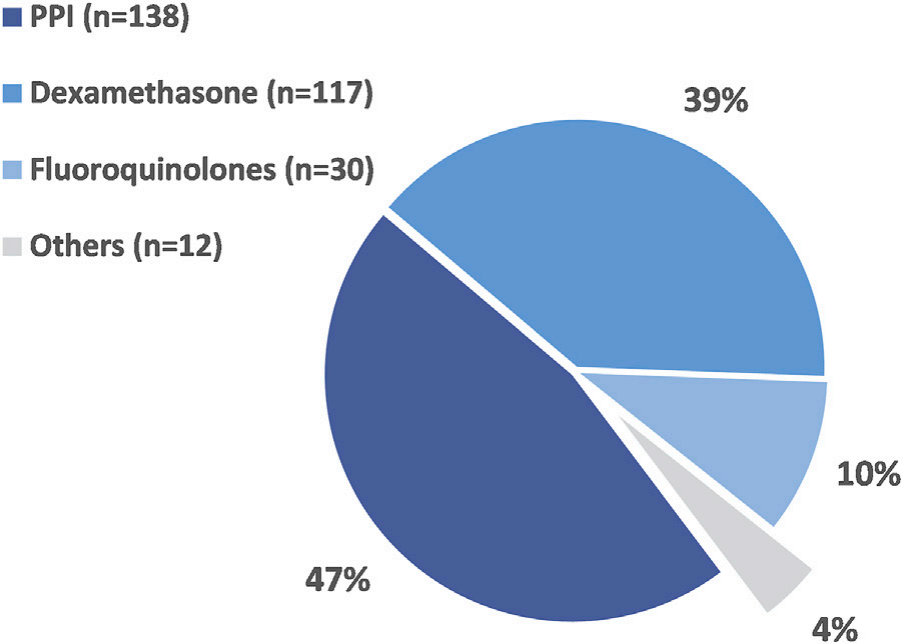
The proportion of different drug classes associated with potentially hazardous DDIs in the studied population.

A total of 297 DDI contraindications were identified in 285 patients (2.0%) considering for oral antineoplastic agent. Pharmacokinetically hazardous DDIs were found in 87.5% of all cases. Potentially hazardous DDIs associated with oral anticancer drugs and other agents are listed in Supplementary Table S3. The class of drugs most frequently associated with hazardous DDIs with oral antineoplastic agents were PPIs (47%), dexamethasone (39%) and fluoroquinolones (10%) (Figure 1). The most common contraindications were the concomitant use of PPIs with certain molecularly targeted agents. PPIs involved in dangerous DDIs with oral chemotherapy may require multiple days of exposure to be clinically relevant. In our study, these long-term drug exposure-related DDIs accounted for 46.5% of all hazardous DDIs. The next most common combination was toremifene, imatinib or axitinib with dexamethasone and potential QTc prolonging oral anticancer drugs with fluoroquinolones or domperidone.

### Potential Risk Factors

All patients were included in the binary logistic regression analysis. In the unadjusted analysis, age, number of comorbidities, number of drugs, number of days hospitalized, tumor type and patients receiving targeted therapy were associated with an increased risk for hazardous DDIs. Supplementary Table S1 shows the results of the univariate and multivariate binary logistic regression analyses. After adjustment for confounders, age [age –020, odds ratio (OR) 0.95 (95% confidence interval (CI) 99.6–1,186.8)], number of drugs [OR 1.08 (95% CI 1.06–1.1)] and treatment type [targeted therapy, OR 4.83 (95% CI 1.45–16.04)] remained statistically significant.

## Discussion

Our study found that PPIs, dexamethasone and fluoroquinolones were the most commonly reported DDIs along with OAAs. Factors such as increased number of medications, younger age, longer hospital stays and targeted therapy are associated with an increased risk of dangerous DDIs. We observed that a prevalence of hazardous DDIs with 2% of all patients being exposed to at least one DDI. The finding that increasing number of medications was a risk factor for potential drug interactions in our population is consistent with the results of previous studies (van Leeuwen et al., 2013). This can be explained by the fact that the number of medications is likely to increase as the length of stay for treatment increases. Remarkably, young age has been identified as a risk factor for contraindicated DDIs. A plausible explanation for this is that patients receiving targeted therapy are much younger than other type of therapy. The highest prevalence was attributed to targeted therapy, accounting for up to 60% of all contraindicated DDIs.

The most commonly used drugs with potentially hazardous interactions with OAAs were PPIs. Patients undergoing cancer treatment often use acid-reducing agents such as PPIs, H_2_-receptors antagonists, and antacids for gastroesophageal reflux disease, dyspepsia, or gastritis associated with chemotherapy (Budha et al., 2012). Acid-reducing agents are prescribed in about 20–33% of cancer patients, and among them, PPI is the most prescribed. However, the use of these agents also increases the risk of potential DDIs, as the dissolution and subsequent absorption of many orally administered, molecularly targeted anticancer drugs exhibit pH-dependent solubility. There are many factors that affect the absorption of anticancer drugs, but pH-dependent solubility is one of the main determinants and the oral bioavailability of these drugs can be significantly altered when administered with PPIs (Smelick et al., 2013; van Leeuwen et al., 2017). The risk of death in cancer patients increased by 16% in combination with various molecularly-targeted anticancer drugs and PPIs therapy (Sharma et al., 2019). In a retrospective study, overall survival in patients with advanced non-small cell lung cancer treated with erlotinib plus acid suppression was significantly different from that of the no-acid-suppression group (12.9 vs. 16.8 months; *p* = 0.003) (Chu et al., 2015). Consequently, the potential for absorption-related drug interactions with these oral targeted oncolytic agents is often observed in hematology/oncology practice, and it is important to understand the impact of these interactions to avoid the reduction of drug efficacy (Budha et al., 2012).

Prolongation of the QTc interval is one of the known but relatively rare side effects of many anticancer drugs including several molecular targeted drugs (including sorafenib, crizotinib and nilotinib) and antihormonal drug (toremifene). These anticancer therapies have properties known to induce QTc interval prolongation through various mechanisms, including direct effects on ion channels and indirectly *via* intracellular signaling pathways (Chandrasekhar and Fradley, 2019). Fluoroquinolones and domperidone are commonly used to treat cancer patients with infections, nausea or vomiting. However, cardiac adverse events such as QTc prolongation and amplified risk of torsades de pointes (TdP) have also been observed in patients taking fluoroquinolones or domperidone (Rossi and Giorgi, 2010; Douros et al., 2015). Although the incidence of cardiac events is low with the use of fluoroquinolones or domperidone alone, the concurrent use of drugs that potentially prolong the QTc interval may markedly increase the risk of pro-arrhythmic effects (Haverkamp et al., 2012; Biewenga et al., 2015; Ehrenpreis et al., 2017; Brunetti et al., 2019). Therefore, the administration of these anticancer drugs should be carefully monitored during concurrent use of other potential QTc prolonging drugs. If possible, an alternative drug that do not affect the QTc interval should be selected (Briasoulis et al., 2011). Electrocardiogram (ECG) monitoring during initiation of these QTc-interval-prolonging anticancer drugs is only necessary in patients who have an underlying condition that predisposes them to TdP or are receiving concomitant medications that may prolong the QTc interval.

Dexamethasone is widely used in the treatment of breast cancer to combat the side effects of chemotherapy and to treat symptoms related to advanced cancer. Although FDA-approved labeling indicates contraindications to the use of toremifene, sorafenib, or imatinib with dexamethasone, it is unclear whether DDIs between dexamethasone and these anticancer drugs has a clinical relevance. Evidence may reflect *in vitro* data indicating that dexamethasone is a relatively weak inducer compared to the prototype inducer and the ligand of the pregnane X receptor activator rifampicin (Revollo and Cidlowski, 2009). In clinical trials, erlotinib is known as a substrate for CYP3A4, and even short-term administration of 4 mg of dexamethasone for 3 days did not affect erlotinib concentrations (Ranson et al., 2010). Moreover, dexamethasone as a weak inducer is not contraindicated on the FDA-approved labeling of other known substrate of CYP3A4 anticancer drug substrates. Therefore, the existence of clinically significant drug interactions between dexamethasone and some anticancer drugs requires further clinical studies.

A major limitation of this study is that the clinical consequences of these drug interactions have not been investigated because of the limited information available in our dataset. We studied DDIs only while patients were hospitalized, and there are currently no studies on whether patients will continue to use them after discharge. It is unclear whether short-term contraindications affect the overall effectiveness of cancer treatment. Another limitation relates to FDA-approved labeling to identify all potentially hazardous drug combinations. Due to the large amount of screening data, we mainly use only the specific drugs listed to determine DDI pairs in the analysis based on the FDA labeling and do not include the entire class of such drugs, which may likely to miss some clinically relevant DDIs for identification. Nevertheless, drug labeling is an important resource that provides detailed information on contraindicated drug combinations information and is an important aid in clinical decision-making. Additionally, several contraindicated drug combinations, including OTC drugs, may have been missed, and herbal medicines used in hospitals could not be identified in this study due to lack of information in computer system records. Some foods containing enzyme inhibitors (including grapefruit juice) were not available in our hospital system, which may significantly affect the metabolism of some OAAs.

In our study, only 2% of the 13,917 patients included in the study were identified using potentially hazardous interacting medicines. However, high-risk patients, such as those receiving targeted therapy or those receiving a growing number of drugs, especially patients taking pH-dependent molecularly targeted agents and potential QTc prolonging drugs, should be concerned about potential drug interactions. To maximize the safety and efficacy of oral antineoplastic agents concurrently with other medications, clinicians and clinical pharmacologists should to be more aware of these potential contraindications in hematology/oncology and work closely to identify and treat these DDIs before the start and during anticancer treatment.

## Data Availability

The raw data supporting the conclusion of this article will be made available by the authors, without undue reservation.
