# Supplementary material for: Potentially Hazardous Drug-Drug Interactions Associated With Oral Antineoplastic Agents Prescribed in Chinese Tertiary Care Teaching Hospital Settings: A Multicenter Cross-Sectional Study
Source: Front Pharmacol. 2022 Feb 1;13:808848. doi: 10.3389/fphar.2022.808848 (PMC8844504; doi:10.3389/fphar.2022.808848)
Supplement: Supplementary file 1 [file DataSheet1.docx]

**Table S1. Oral antineoplastic agents evaluated in the retrospective study**

| **OAAs Subclass** | **Agents** |
| --- | --- |
| Molecular targeted agents | afatinib, alectinib, axitinib, crizotinib, dasatinib, erlotinib, gefitinib, ibrutinib, imatinib, ixazomib, lapatinib, nilotinib, olaparib, osimertinib, pazopanib, sorafenib, sunitinib, regorafenib, ruxolitinib, vemurafenib |
|  |  |
| Antihormonal agents | anastrozole, bicalutamide, abiraterone, tamoxifen, toremifene, letrozole, exemestane |
| Cytotoxic agents | methotrexate, capecitabine, temozolomide |

OAAs, Oral antineoplastic agents

**Table S2. Characteristics of patients in the study^a^**

| **Patient characteristic** | **No. of patients or value** | **%** |
| --- | --- | --- |
| Study population | 13917 | 100 |
| Age | 57 (4-95) | - |
| No. of drugs | 7 (1-63) | - |
| No. of hospital days | 5 (1-90) | - |
| No. of comorbidities | 0.5 (0-8) | - |
| Sex |  |  |
| *Male* | 4616 | 33.17 |
| *Female* | 9301 | 66.83 |
| Tumor type |  |  |
| *Solid malignancy* | 13459 | 96.71 |
| *Haemato-oncology* | 458 | 3.29 |
| Cancer-type solid malignancy |  |  |
| *Lung* | 757 | 5.44 |
| *Breast* | 6769 | 48.64 |
| *Liver* | 214 | 1.54 |
| *Other* | 5719 | 41.09 |
| Cancer-type haemato-oncology |  |  |
| *Malignant lymphoma* | 218 | 1.57 |
| *Plasma cell dyscrasia* | 185 | 1.33 |
| *Leukemia* | 49 | 0.35 |
| *Myelodysplastic syndrome* | 6 | 0.04 |

^a^, Values are median (range) or n (%).

**Table S3. List of contraindicated oral antineoplastic drugs and other medications**

| **OAAs** | **Contraindicated medication** | **Mechanism** | **Description** | **n** |
| --- | --- | --- | --- | --- |
| Gefitinib | PPI | Pharmacokinetic | Drug combinations may decrease the plasma concentration of gefitinib | 107 |
| Toremifene | Dexamethasone | Pharmacokinetic | Drug combinations may decrease the plasma concentration of toremifene | 99 |
|  | Fluoroquinolones | Pharmacodynamic | Drug combinations may prolong QT interval | 5 |
| Dasatinib | PPI | Pharmacokinetic | Drug combinations may decrease the plasma concentrations of dasatinib | 22 |
| Imatinib | Dexamethasone | Pharmacokinetic | Drug combinations may decrease the plasma concentrations of imatinib | 16 |
| Crizotinib | Fluoroquinolones | Pharmacodynamic | Drug combinations may prolong QT interval | 11 |
|  | Domperidone | Pharmacodynamic | Drug combinations may prolong QT interval | 4 |
| Osimertinib | Fluoroquinolones | Pharmacodynamic | Drug combinations may prolong QT interval | 8 |
|  | Domperidone | Pharmacodynamic | Drug combinations may prolong QT interval | 2 |
| Erlotinib | PPI | Pharmacokinetic | Drug combinations may decrease the plasma concentration of erlotinib | 7 |
| Nilotinib | Fluoroquinolones | Pharmacodynamic | Drug combinations may prolong QT interval | 3 |
|  | PPI | Pharmacokinetic | Drug combinations may decrease the plasma concentration of nilotinib | 2 |
|  | Voriconazole | Pharmacokinetic | Drug combinations may increase the plasma concentration of nilotinib | 2 |
|  | Domperidone | Pharmacodynamic | Drug combinations may prolong QT interval | 1 |
| Sorafenib | Fluoroquinolones | Pharmacodynamic | Drug combinations may prolong QT interval | 3 |
| Lapatinib | Phenobarbital | Pharmacokinetic | Drug combinations may decrease plasma concentrations of lapatinib | 2 |
|  | Voriconazole | Pharmacokinetic | Drug combinations may increase the plasma concentration of lapatinib | 1 |
| Axitinib | Dexamethasone | Pharmacokinetic | Drug combinations may decrease the plasma concentrations of axitinib | 2 |

OAAs, Oral antineoplastic agents; PPI, Proton pump inhibitor.

**Table S4. Univariate and multivariable-adjusted analyses of potential drug interactions**

| **Variable** | **Unadjusted OR (95% CI)** | **Unadjusted *P* value** | **Adjusted OR (95% CI)** | **Adjusted *P* value** |
| --- | --- | --- | --- | --- |
| Age ranges |  |  |  |  |
| *Age: 41-60* | 1 (Ref.) |  |  |  |
| *Age:0-20* | 53 (18.9 to148.5) | <.0001 | 343.89 (99.64 to1186.84) | <.0001 |
| *Age:21-40* | 2.03 (0.7 to5.9) | 0.19 | 2.28 (0.81 to6.45) | 0.12 |
| *Age:61-80* | 1.85 (0.7 to5.0) | 0.23 | 2.27 (0.74to6.93) | 0.15 |
| *Age:≥81* | 1.17 (0.4 to3.2) | 0.76 | 1.1 (0.39 to3.14) | 0.86 |
| No. of comorbidities | 1.38 (1.3 to 1.51) | <.0001 | 0.98 (0.87 to1.1) | 0.67 |
| No. of drugs | 1.08 (1.07 to 1.09) | <.0001 | 1.08 (1.06 to1.1) | <.0001 |
| No. of hospital days | 1.07 (1.05 to 1.08) | <.0001 | 1.0 (0.98 to1.03) | 0.66 |
| Tumor type |  |  |  |  |
| *Solid malignancy* | 1 (Ref.) |  |  |  |
| *Haemato-oncology* | 5.16 (3.64 to 7.31) | <.0001 | 1.43 (0.89 to2.92) | 0.13 |
| Treatment type |  |  |  |  |
| *Antihormonal therapy* | 1 (Ref.) |  |  |  |
| *Targeted therapy* | 4.37 (1.37 to 13.92) | 0.013 | 7.02 (1.42 to34.78) | 0.017 |
| *Chemotherapy* | 0 | 0.97 | 0 | 0.97 |
| *Combination* | 0.53 (0.17 to 2.0) | 0.29 | 1.19 (0.24 to5.95) | 0.83 |
